# Seed disperser connectivity in a heterogeneous landscape of the Colombian Coffee Region

**DOI:** 10.1371/journal.pone.0351834

**Published:** 2026-06-26

**Authors:** Isabel Cristina Restrepo-Carvajal, Nicola Clerici, Swanni T. Alvarado

**Affiliations:** 1 School of Sciences and Engineering, Universidad del Rosario, Bogotá D.C., Colombia; 2 Departamento de Biología, Facultad de Ciencias, Universidad Nacional de Colombia, Bogotá D.C., Colombia; UNAM, MEXICO

## Abstract

Habitat fragmentation is a major threat to biodiversity in tropical montane ecosystems, where limited forest cover and steep elevational gradients can strongly constrain species movement and connectivity between habitat fragments. We assessed landscape fragmentation, functional connectivity of habitat patches for four seed-dispersing mammals (i.e., the capacity of species to move among habitat patches), and conservation priorities in the Colombian Coffee Region, a highly transformed Andean landscape. Using resistance-based distance matrices, graph-based landscape connectivity indices (PC, dPC, and ECA) and sensitivity analyses across resistance and dispersal scenarios we quantified species-specific connectivity, identified key habitat nodes, and evaluated their representation within the current protected area network. We also incorporated uncertainty into a multi-species prioritization framework to improve conservation decision-making. Forest cover is highly reduced (remains 18.9% of its original extent) and severely fragmented, resulting in low functional connectivity to all species. Connectivity patterns were primarily associated with species’ elevational range rather than home range size, with generalist species maintaining higher connectivity than species restricted to narrower elevations. Connectivity was highly uneven, with a small number of habitat patches acting as critical network hubs whose loss could substantially reduce overall connectivity. Despite their importance, more than half of the total habitat and many high-priority connectivity areas remain outside protected areas, revealing a strong mismatch between conservation priorities and current protection coverage. Incorporating uncertainty further refines prioritization by distinguishing robust conservation targets from areas requiring additional empirical validation. Our results demonstrate that integrating multi-species connectivity modeling with uncertainty analysis provides a robust framework for identifying conservation priorities. These findings are particularly relevant for vulnerable and range-restricted species such as *Cebus versicolor* and emphasize the need for restoration and connectivity-oriented conservation strategies in human-modified tropical landscapes.

## Introduction

Habitat fragmentation is a major driver of biodiversity loss [1], particularly in tropical montane ecosystems where land-use change has transformed continuous forests into heterogeneous mosaics of habitat and non-habitat areas [2]. Beyond reducing total habitat area, fragmentation disrupts ecological processes by decreasing functional connectivity [3], i.e., the degree to which landscapes facilitate or impede movement among habitat patches [4]. Functional connectivity depends not only on the spatial arrangement of habitat remnants, but also on matrix resistance and species-specific movement capacities [4]. In fragmented landscapes, habitat patches are embedded within a non-habitat matrix that varies in resistance to movement [5]. Matrix resistance reflects the difficulty that organisms experience when moving through different land-cover types, thereby influencing dispersal success and overall connectivity [4]. Graph-based connectivity indices provide a computationally efficient framework for analyzing these dispersal success and overall connectivity by representing suitable habitat patches as nodes and potential dispersal pathways as links within a network [6]. Links may represent physical corridors or functional connections derived from Euclidean or minimum-cost distances that account for species movement preferences across heterogeneous land-cover types [7].

Fragmentation processes are especially critical in mountain systems, where steep environmental gradients and naturally restricted elevational distributions constrain the movement of many species across the landscape [8]. As a result, montane species are particularly vulnerable to habitat loss and isolation, since many occupy narrow climatic and altitudinal ranges and depend on habitat continuity along elevation gradients to maintain dispersal, gene flow, and access to resources [8]. This vulnerability is particularly pronounced in tropical Andean ecosystems, where high levels of endemism combined with ongoing landscape transformation increase the risk of population isolation, local extinctions, and disruption of key ecological processes [9].

In the tropical Andes, and particularly within Colombia’s Coffee Region, forest cover has been extensively reduced and fragmented by agricultural expansion [10]. This region represents a highly transformed socio-ecological system where conservation actions must balance biodiversity protection with productive land uses [11]. In this context, identifying areas that are critical for maintaining connectivity is essential for guiding conservation planning, especially where the expansion of protected areas is constrained by socio-economic limitations [12].

Animal-mediated seed dispersal is a key ecological process linking landscape connectivity to ecosystem functioning [13]. Many tropical tree species, particularly those producing medium- and large-sized seeds, depend on vertebrates for dispersal, as abiotic mechanisms are often insufficient for effective seed movement [14]. The persistence of these plant species —and the ecosystem services they support— relies on the ability of dispersers to move seeds across heterogeneous landscapes, facilitating gene flow, recruitment, and spatial population dynamics [13]. Disruptions to connectivity may therefore generate downstream effects on forest regeneration, biodiversity maintenance and carbon storage.

This study focuses on four mammal species with complementary roles in seed dispersal: *Alouatta seniculus*, *Cebus versicolor*, *Cuniculus paca*, and *Dasyprocta punctata*. Frugivorous primates such as *A. seniculus* and *C. versicolor* typically disperse seeds over relatively long distances through daily movements, contributing to connectivity among plant populations [15]. In contrast, large rodents such as *C. paca* and *D. punctata* act as both seed predators and dispersers via scatter-hoarding behavior, influencing seed survival and establishment [16]. These differences in dispersal mode, combined with variation in movement capacity, habitat use, and altitudinal distribution, provide complementary perspectives on how functional connectivity shapes seed dispersal processes across the landscape. The objectives of this study are: i) to characterize landscape fragmentation in the Colombian Coffee Region; ii) to quantify species-specific functional connectivity using resistance-based metrics; and iii) to identify and prioritize habitat nodes that contribute most to maintaining multi-species connectivity, and to assess their representation within existing protected areas.

## Methods

### Study area

**Fig 1:**
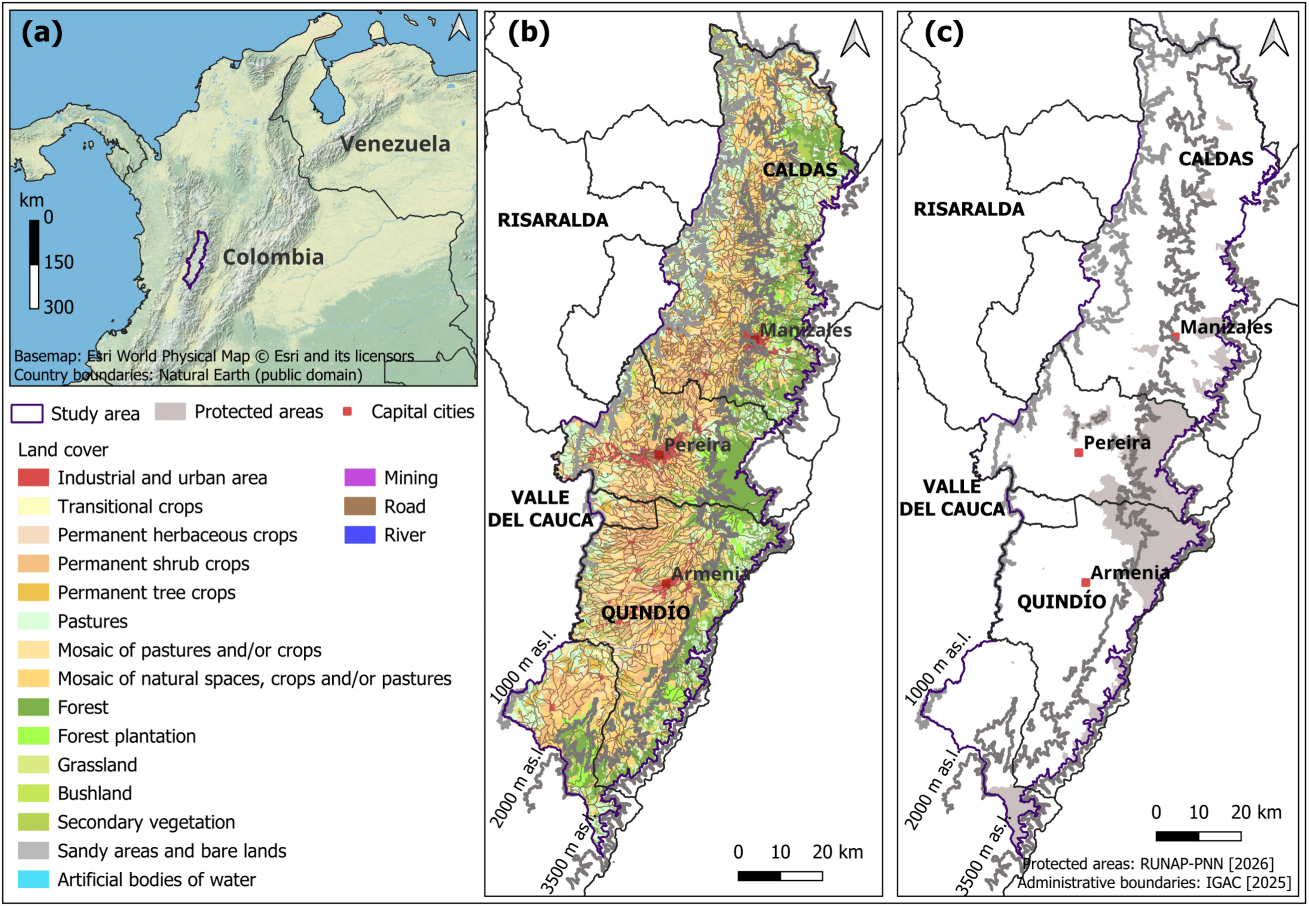
a) Location in Colombia, b) Land Use/Land Cover, c) protected areas and c) coffee cultural landscape on the study area.

The study was conducted on the western slopes of Colombia’s Central Andean cordillera, covering 6,294.43 km^2^ across the departments of Caldas, Risaralda, Quindío, and northern Valle del Cauca (Fig 1). Elevation ranges from ~600–3,200 m a.s.l., encompassing Sub-Andean (≤2,000 m a.s.l.) and High Andean forests (2,000–3,200 m a.s.l.) [10]. The region, originally dominated by moist forests, is now a heterogeneous mosaic of land uses, including coffee, banana, citrus and avocado agricultural systems, pastures, rural settlements, and urban areas, with major cities such as Armenia, Pereira, and Manizales (Fig 1a) [17]. Temperature ranges from 12 to 30 °C and annual precipitation from 1,487–7,226 mm [18]. A total of 905.7 km^2^ of the study area is legally protected within the National System of Protected Areas (Fig 1b) [19].

The study area was delimited to include Sub-Andean and High Andean forests, the suitable habitat for the four focal species on the region. The eastern (upper) boundary was set at 3,200 m a.s.l., corresponding to the maximum altitudinal distribution of both the species and the High Andean forests [10], while the western (lower) boundary is defined by the Cauca River and its tributary, the La Vieja River, which act as effective dispersal barriers due to their high discharge. The northern and southern limits follow the political boundaries of the department of Caldas (north) and the municipality of Sevilla, Valle del Cauca (south), corresponding to the core extent of the Coffee Cultural Landscape, an UNESCO recognition of the coffee farming traditions, cultural practices, and natural environment distinctive of the region [20].

### Fragmentation metrics

To characterize the structural condition of the landscape and provide a baseline for interpreting functional connectivity patterns, we calculated a set of fragmentation metrics at both landscape and patch levels. At the landscape level, we quantified the total forest area as an indicator of habitat availability, and the number of patches and mean patch size as measures of fragmentation. We also calculated the total core area (defined as habitat unaffected by a 30 m edge effect, following [21]) to identify the amount of interior habitat available. To explicitly assess functional habitat availability, we calculated the number of patches with area smaller than 0.01 km^2^, corresponding to the minimum home range size of the focal species. These patches are unlikely to support viable populations and cannot be considered habitat for the focal species.

At the patch level, we analyzed the distribution of key attributes including patch area, core area, and shape index to characterize variability in habitat structure across the landscape. Histograms of area and core area were log-transformed (log₁₀) to improve visualization of their distribution. All analyses and visualizations were performed using the Makurhini package [22] in RStudio (version 2026.04.0 + 526) [23].

To assess how forest distribution varied with elevation, the study area was divided into lower and upper elevation zones at 2,000 m a.s.l., a threshold close to the transition belt between Sub-Andean and High Andean forests [10]. This threshold also approximates the upper altitudinal limit of *D. punctata* and *C. versicolor*. The lower elevation area (0–2,000 m as. l.) covers 4,201.86 km^2^, while the upper area (2,000–3,200 m as. l.) covers 2,096.47 km^2^.

### Focal species

Four terrestrial mammal species with key roles in seed dispersal were selected as focal species: the white-fronted capuchin (*Cebus versicolor*), red howler monkey (*Alouatta seniculus*), lowland paca (*Cuniculus paca*), and Central American agouti (*Dasyprocta punctata*). These species are known to inhabit the Colombian Andes and have been reported in the coffee-growing region [24,25]. Their ecological traits—particularly related to movement capacities, habitat use, and sensitivity to landscape fragmentation—make them valuable indicators and targets for assessing functional connectivity in human-modified landscapes.

*C. versicolor*, a generalist and behaviorally flexible primate, exhibits strong reliance on forested areas but may tolerate moderate levels of fragmentation [26]; its reported home range vary from 120 ha in conserved ecosystems [26] to 30 ha in fragmented ecosystems in Colombia [27] and may inhabit forests from sea level to 2000 m as.l. [28]. A*. seniculus* is a primarily arboreal, folivorous-frugivorous primate with a home range estimated between 10–17 ha in the Magdalena Valley in Colombia [29]. A*. seniculus* inhabits forests from sea level to 3200 m a.s.l. [30] and its movement is highly dependent on the continuity of forest canopies [26].

The two rodent species—*C. paca* and *D. punctata*—are primarily terrestrial seed dispersers. *C. paca* is nocturnal, prefers dense understory forests for cover, and typically occupies home ranges of 1–3 ha [31]; its altitudinal presence varies from 0 to 2300 m a.s.l. [32]. *D. punctata* is diurnal, more active, and wide-ranging, with home ranges varying between 1–6 ha [33], is known to be more tolerant of fragmented and human-altered landscapes [34] and is distributed from 0 to 1500 m a.s.l [35].

Species presence within the study area was confirmed using GBIF occurrence records from the past ten years [28,29,32,35]. All analyses were conducted using secondary spatial datasets across a broad regional extent; therefore, field access permits were not required. Ethical approval was granted by the Life Sciences Committee of the Research Ethics Board at Universidad del Rosario (approval code DVO005 2866 - CV1870, March 18, 2025).

### Resistance surface and suitable habitat identification

Species-specific resistance surfaces were generated to represent movement difficulty across the landscape. A 30 m resolution digital elevation model was first used to constrain analyses to the altitudinal range of each species. Land cover data were obtained from the 2020 CORINE Land Cover dataset for Colombia (100 m resolution) [17], and road networks from the National Cartography database (100 m resolution) [36]. The CORINE Land cover layer was reclassified and combined with the road network 19 land-cover categories. Resistance values were assigned based on published ecological information and expert knowledge (detailed parametrization explained in S1 Methods), reflecting species-specific responses to different land-cover types and anthropogenic features. To account for uncertainty, three resistance scenarios were defined: (1) low contrast, with reduced differences among land-cover classes; (2) baseline, representing intermediate resistance values; and (3) high contrast, with increased resistance assigned to non-forest areas (Resistance values for each scenario in S1 Table).

Habitat nodes were defined as forest patches having a 30 m edge buffer [21] and excluding patches whose core area was smaller than the minimum reported home range of each species (Table 1).

**Table 1:** Reported home ranges for the studied species with its sources, minimum area defined for habitat patches and altitudinal distribution for the studied species with its sources.

Species	Home range (km²)	Source	Minimum area defined for habitat nodes (km²)	Altitudinal distribution (m as.l.)	Source
*Cebus versicolor*	0.30-1.20	[26,37]	0,30	0–2,000	[28]
*Alouatta seniculus*	0.10-0.17	[29]	0,10	0–3,200	[30]
*Cuniculus paca*	0.01–0.03	[38]	0,01	0–2,300	[32]
*Dasyprocta punctata*	0.01-0.06	[33]	0,01	0–1,500	[35]

### Dispersal parameterization

Species dispersal capacity was estimated based on home range size, using the equations presented at [31]. To represent realistic dispersal behavior, a log-normal dispersal kernel was parameterized for each species, capturing the fat-tailed nature of dispersal observed in ecological systems [39]. To reduce computational complexity, each kernel was approximated using five representative distances corresponding to the 10th, 25th, 50th, 75th, and 95th percentiles. This discrete approximation preserves both short- and long-distance dispersal processes while enabling efficient iterative modeling [40].

To incorporate resistance surface into dispersal, Euclidean dispersal distances (the five representative distances) were transformed into effective dispersal distances by scaling them using the mean resistance of the landscape matrix. Mean resistance was calculated as the arithmetic mean of raster values (excluding missing data), thereby weighting resistance by spatial extent (Full data extents in S2 Table).

### Functional connectivity and uncertainty analysis

Functional connectivity was quantified using least-cost distance matrices derived from species-specific resistance surfaces. These matrices integrate inter-patch distance and landscape resistance to estimate movement probabilities between habitat nodes. We calculated two complementary connectivity metrics: (1) Probability of Connectivity (PC) (Equation 1a) [41], which quantifies the probability that two individuals randomly located within habitat patches can reach each other; (2) Equivalent Connected Area (ECA), which expresses connectivity in area units as the size of a single, fully connected habitat patch providing the same connectivity as the observed landscape (Equation 1b) [42].

PC was calculated as:


$$\begin{array}{c} PC=\frac{\text{1}}{\overset{\text{2}}{\underset{L}{A}}}\sum \overset{n}{\underset{i=\text{1}}{\mathrm{\backslash nolimits}}}\sum \overset{n}{\underset{j=\text{1}}{\mathrm{\backslash nolimits}}}a_{i}a_{j}p_{ij} \end{array}$$ (1)

ECA was calculated as:


$$\begin{array}{c} ECA=\sqrt{\sum \overset{n}{\underset{i=\text{1}}{\mathrm{\backslash nolimits}}}\sum \overset{n}{\underset{j=\text{1}}{\mathrm{\backslash nolimits}}}a_{i}a_{j}p_{ij}} \end{array}$$ (2)

Where *A*_*L*_ (1) is the total landscape area, *a*_*i*_ and *a*_*j*_ represent the area of habitat patches *i* and *j*, and *p*_*ij*_ is the probability of dispersal between patches *i* and *j* which depends on inter-patch distance and landscape resistance.

To assess the contribution of individual habitat nodes to functional connectivity, we calculated the relative change in PC values (dPCk) following the removal of each node. These metrics quantify the reduction in habitat availability caused by the absence of a specific patch, incorporating its area, its role in supporting dispersal, and its function as a stepping stone. dPCk was further decomposed into three additive components: intra (internal habitat area), flux (direct dispersal contribution), and connector (stepping-stone role in indirect connections) representing the distinct ways in which a node contributes to functional connectivity (Equation 3) [see 43].


$$\begin{array}{c} {\text{dPC}}_{\text{k}}\;=\;{\text{DPCintra}}_{\text{k}}\;+\;{\text{DPCflux}}_{\text{k}}\;+\;{\text{DPCconnector}}_{\text{k}} \end{array}$$ (3)

Each resistance scenario (low contrast, baseline, and high contrast) was combined with each dispersal percentile (10th, 25th, 50th, 75th, and 95th) resulting in 15 scenarios per species. Connectivity metrics (PC, ECA, and dPCₖ) were computed using the Makurhini package [22] for each scenario. Within each scenario, connectivity metrics were summarized using probability-weighted means based on dispersal percentiles (weights: 0.1, 0.15, 0.25, 0.25, and 0.2 for the 10th, 25th, 50th, 75th, and 95th percentiles, respectively). These scenario-specific weighted means were then averaged with equal weighting across scenarios to derive overall estimates and between-scenario variance. All analyses and visualizations were performed using the Makurhini package [22] in RStudio (version 2026.04.0 + 526) [23].

### Multi-species prioritization and protection status assessment

Node-level functional connectivity importance (dPC) was quantified independently for each species and subsequently standardized to ensure comparability among taxa with differing dispersal capacities and network structures. For each species, weighted mean dPC values —previously derived by integrating dispersal-distance percentiles and matrix permeability scenarios— were normalized by dividing each node value by the maximum observed dPC. This procedure yielded a relative importance index ranging from 0 to 1.

To integrate species-specific results, all outputs were rasterized to a common 30x30 m grid. Multi-species connectivity was calculated as the arithmetic mean of standardized values across species. These values were classified into three categories of importance (low, medium, high) using percentile thresholds (0–50%, 50–75%, 75–100%). Uncertainty was quantified by propagating species-specific variability. Standard deviations were squared to obtain variances, averaged across species, and square-rooted to derive the multi-species standard deviation. The coefficient of variation (CV) was calculated as the ratio of standard deviation to mean connectivity. Reliability was classified as high (CV < 0.2), moderate (0.2–0.5), or low (> 0.5).

The multi-species connectivity importance layer was overlaid with the national protected areas dataset [19] to quantify the proportion of high-priority areas currently protected and to identify high-importance areas outside protected areas as conservation gaps. Reliability classes were used to distinguish robust from uncertain connectivity estimates.

## Results

### Fragmentation metrics

**Fig 2:**
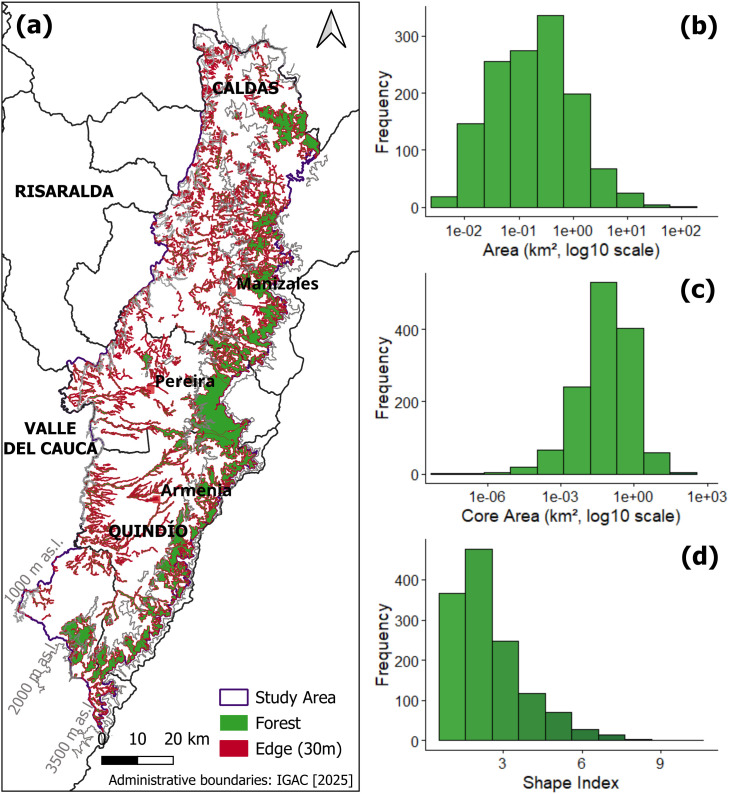
(a) Forest patches (green) and edges (red). (b,c) Log₁₀ distributions of patch area and core area, and (d) distributions of shape index.

Forest patches occupy 18.9% (1,191.37 km^2^) of the study area (Fig 2a), comprising 2,018 patches with a mean patch size of 0.59 + − 3.39 km^2^ (SD). Of these, 722 patches (35.78%) fall below the minimum threshold of 0.01 km^2^. Total core area is 905.75 km^2^, representing 14.39% of the landscape. Forest cover is concentrated at higher elevations, with 778.09 km^2^ (37.11%) above 2,000 m a.s.l. and 412.72 km^2^ (9.82%) below this threshold. Patch size and core area distributions are strongly right-skewed on a log₁₀ scale (Fig 2b), with most values concentrated in smaller classes. Shape index values are left-skewed, with most observations between 1.0 and 2.5.

### Functional connectivity

Habitat node configuration varies among species according to home range size and altitudinal distribution. *C. versicolor* exhibits the lowest number of nodes (190), covering 157.27 km^2^ (3.7% of its range). In contrast, *A. seniculus* shows broader coverage (710 nodes; 865.85 km^2^; 13.8%). *C. paca* presents the highest node density (1,422 nodes; 378.43 km²; 7.7%), while *D. punctata* includes 856 nodes covering 134.81 km^2^ (5.1%).

**Fig 3:**
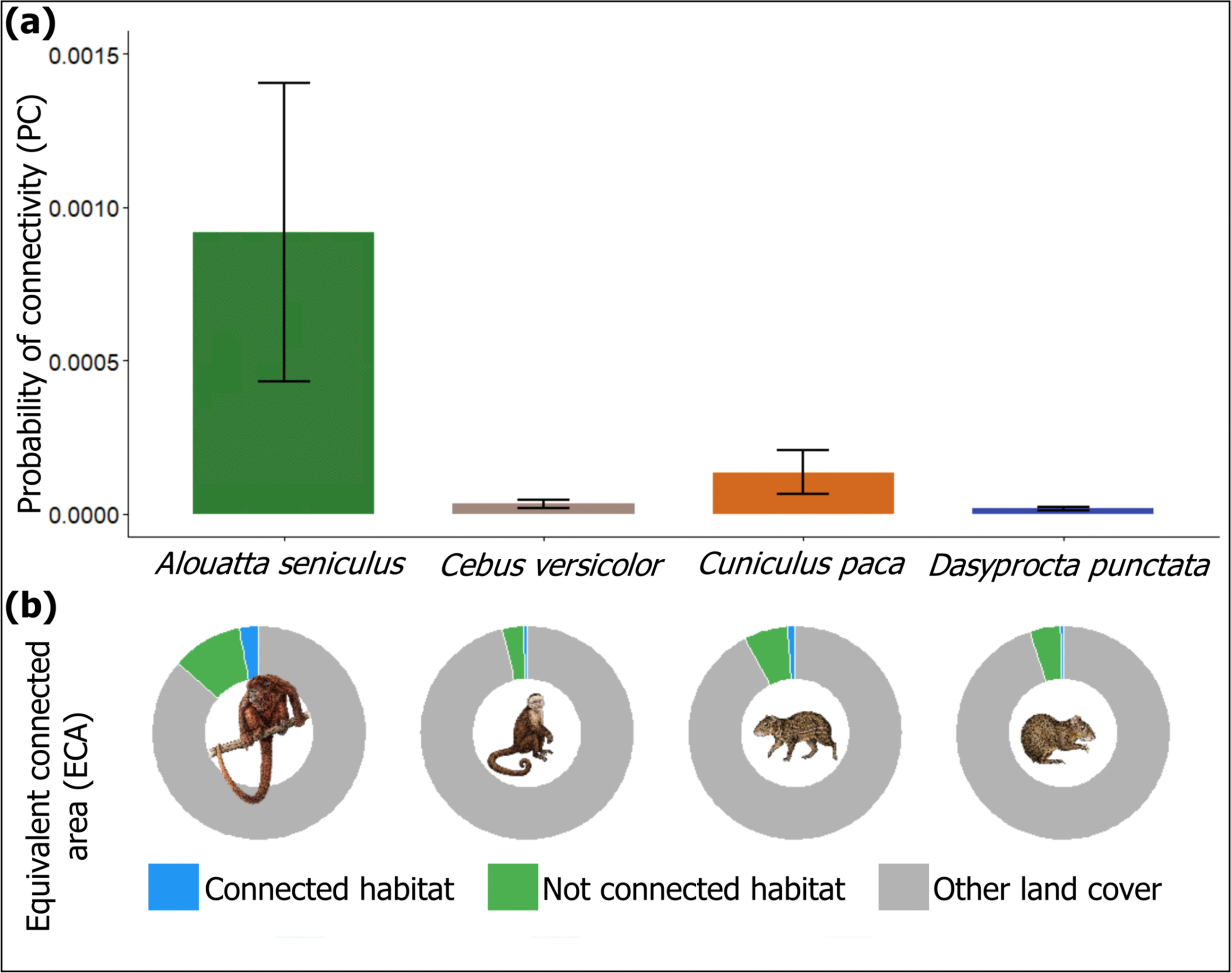
Probability of Connectivity (PC) and Equivalent Connected Area (ECA) across species.

Connectivity metrics reflect differences in habitat configuration among species. The highest PC is observed for *A. seniculus* (0.000917 ± 0.000487 SD), followed by *C. paca* (0.000135 ± 0.000071 SD), *C. versicolor* (0.000031 ± 0.000015 SD), and *D. punctata* (0.000016 ± 0.000005 SD) (Fig 3b). ECA shows the same ranking, with values of 186.3 ± 45.5 km^2^ (*A. seniculus*), 56.0 ± 12.4 km^2^ (*C. paca*), 23.1 ± 5.0 km^2^ (*C. versicolor*), and 10.6 ± 1.7 km^2^ (*D. punctata*). ECA represents 21.51%, 14.79%, 14.69%, and 7.83% of total habitat, respectively, and 2.96%, 1.14%, 0.55%, and 0.40% of the total landscape (Fig 3b).

**Fig 4:**
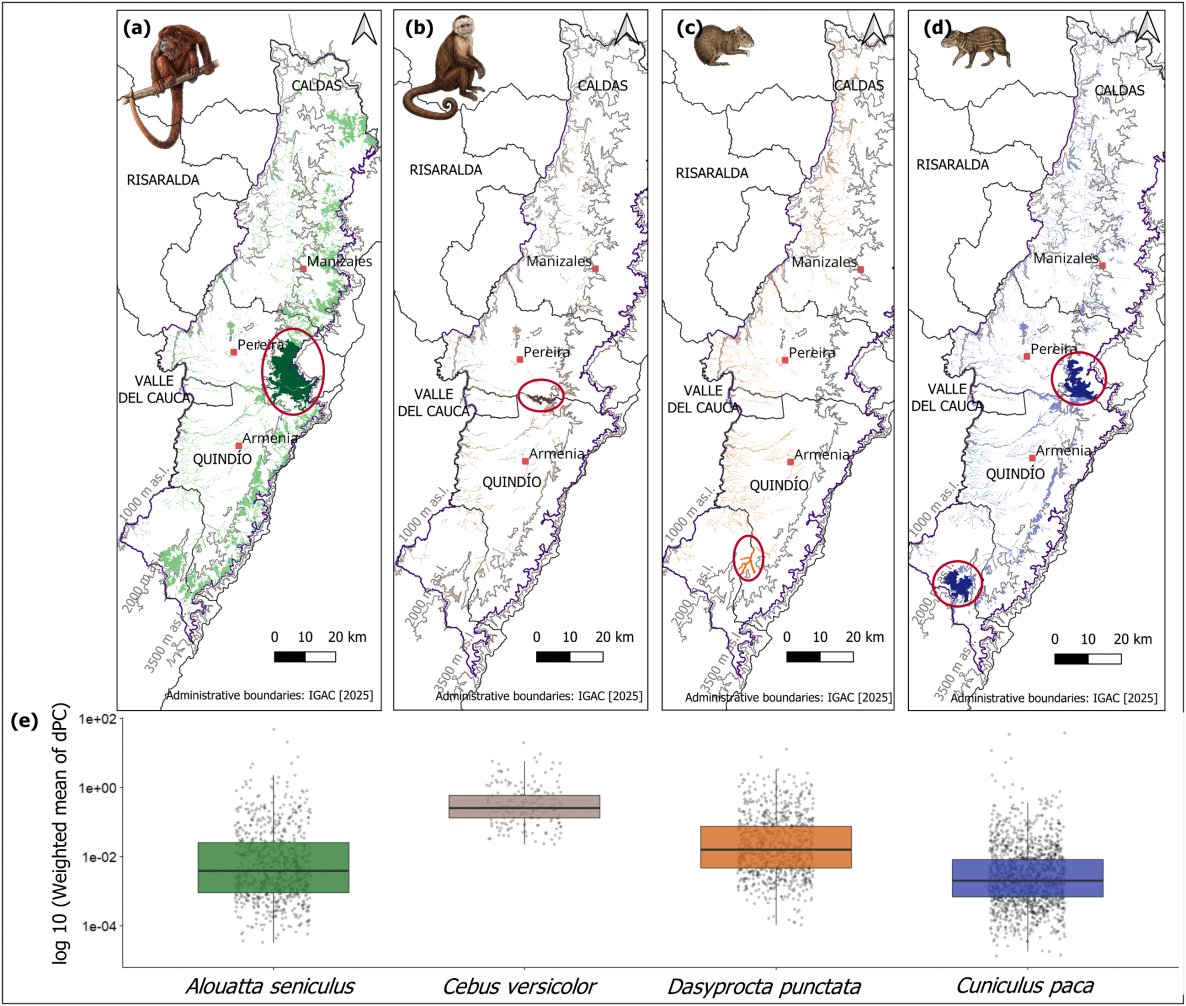
Spatial distribution (a-d) and variability of habitats nodes importance (e) on functional connectivity (dPC) across species. Nodes highlighted with red circles and darker colors represent the main contributors to connectivity (dPC > 10).

Node importance (dPC) values are strongly skewed toward low contributions across species, with most nodes having dPC < 1. *C. versicolor* shows the highest median (0.25 ± 1.82 SD), with 157 of 190 nodes below this threshold and a single highly influential node (dPC = 19.08) (Fig 4b). *D. punctata* shows intermediate values (median = 0.02 ± 0.58 SD), with 829 of 856 nodes with dPC < 1 and one highly influential node (dPC = 12.38) (Fig 4c). In contrast, *A. seniculus* (median = 0.0039 ± 2.00 SD) and *C. paca* (median = 0.0021 ± 1.38 SD) exhibit lower central values and a higher proportion of nodes with dPC < 1. *A. seniculus* includes 694 such nodes and two highly important nodes (dPC = 48.06 and 19.90) (Fig 4a). *C. paca* is similarly dominated by low values (1,413 nodes with dPC < 1), with three nodes exceeding dPC = 10 (36.70, 34.09, and 11.25) (Fig 4d). Uncertainty in dPC values, expressed as the coefficient of variation (CV), differs among species. *C. paca* shows the highest variability (median CV = 1.56 ± 0.76 SD), followed by *A. seniculus* (0.67 ± 0.47 SD) and *D. punctata* (0.54 ± 0.33 SD), while *C. versicolor* presents the lowest variability (0.26 ± 0.21 SD).

**Fig 5:**
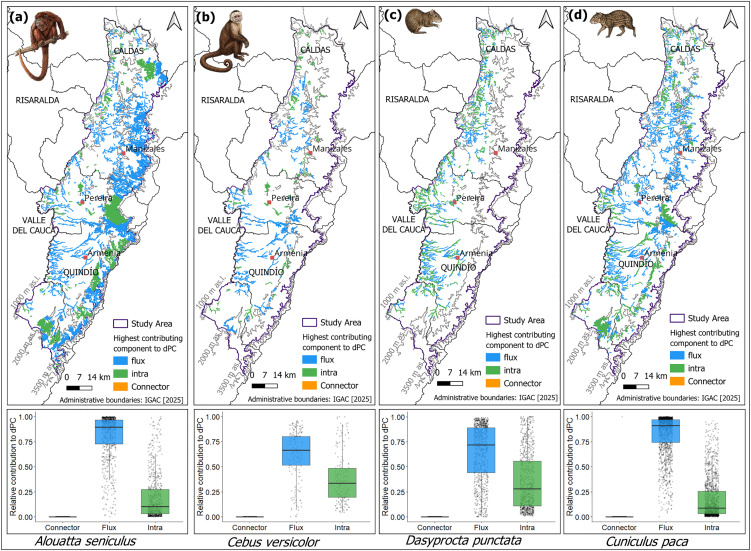
Relative contribution of each component (intra, flux, and connector) to dPC for each species. Upper panels show habitat nodes colored according to the highest contributing component to dPC at each node. Bottom boxplots summarize the relative contribution of each component (intra, flux, and connector) to nodes dPC values.

Differences in the relative contribution of dPC components (intra, flux, and connector) were consistent across species (Fig 5). The connector component is negligible in all cases (median ± SD: *A. seniculus* = 6.85 × 10^−7^ ± 1.02 × 10^−5^; *C. versicolor* = 1.23 × 10^−8^ ± 2.84 × 10^−8^; *D. punctata* = 2.01 × 10^−7^ ± 3.20 × 10^−6^; *C. paca* = 1.69 × 10^−6^ ± 2.65 × 10^−2^) and is the dominant component in only one node (*C. paca*). The flux component was the most important in the majority of nodes across all species (*A. seniculus*: 628/710, median = 8.95 × 10^−5^ ± 2.32 × 10^−5^ SD; *C. versicolor*: 145/190, median = 6.63 × 10^−5^ ± 2.49 × 10^−5^ SD; *D. punctata*: 602/856, median = 7.18 × 10^−5^ ± 2.84 × 10^−5^ SD; *C. paca*: 1,277/1,422, median = 9.11 × 10^−5^ ± 2.08 × 10^−5^ SD), while the intra component is the most important in a smaller proportion of nodes (*A. seniculus*: 83 nodes, median = 1.05 × 10^−5^ ± 2.32 × 10^−5^ SD; *C. versicolor*: 45 nodes, median = 3.37 × 10^−5^ ± 2.49 × 10^−5^ SD; *D. punctata*: 254 nodes, median = 2.82 × 10^−5^ ± 2.84 × 10^−5^ SD; *C. paca*: 144 nodes, median = 8.89 × 10^−6^ ± 2.07 × 10^−5^ SD). These patterns are also spatially consistent, with flux-driven nodes prevailing across the landscape.

### Multi-species conservation priority areas and protection status

**Fig 6:**
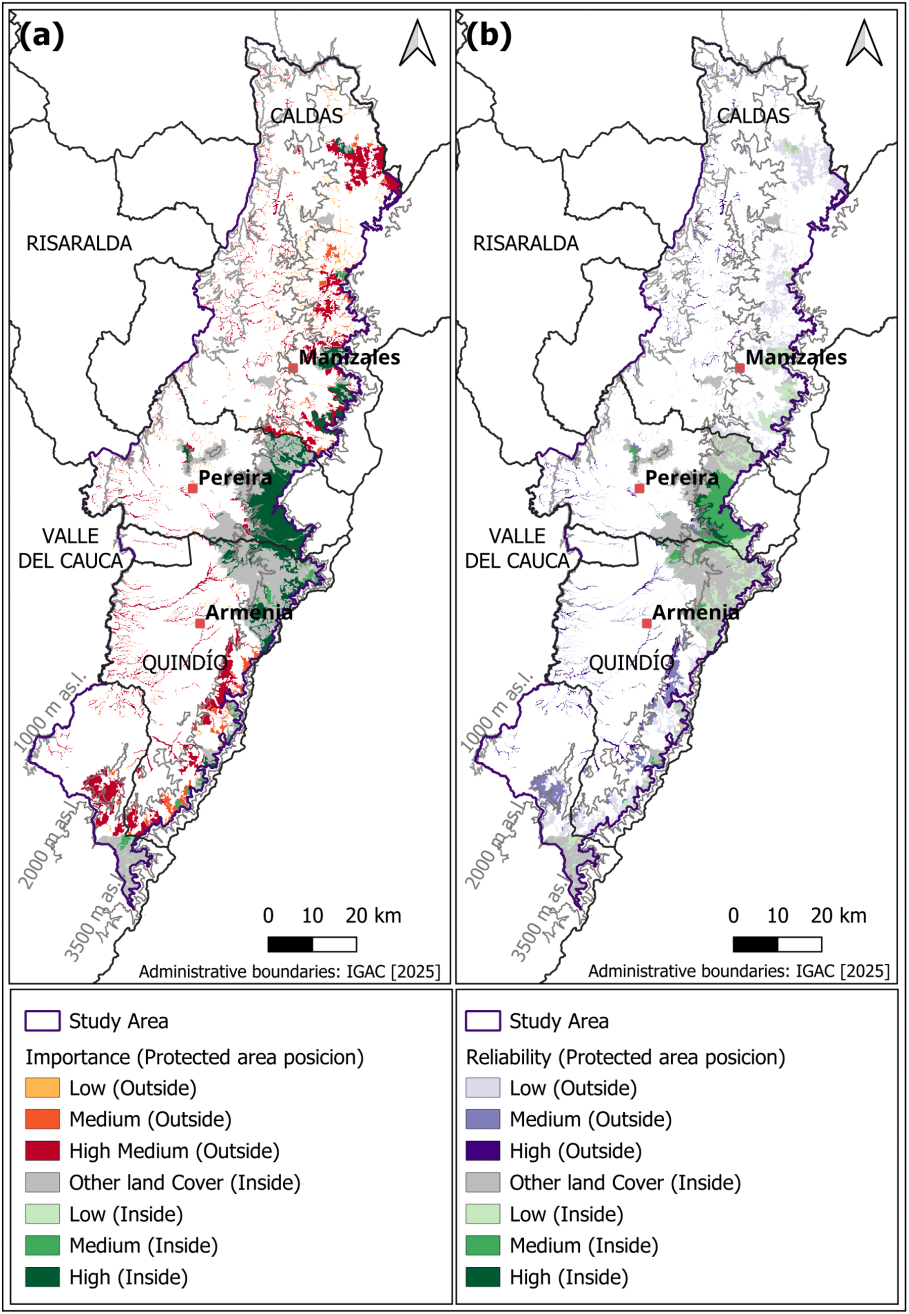
Multi-species connectivity importance, reliability, and protection status. **(a)** Overlay of importance connectivity areas and protected areas **(b)** Overlay of reliability and protected areas.

The multi-species connectivity prioritization identified a total of 896.00 km^2^ of habitat within the study area. Of this, 343.42 km^2^ (38.33%) overlap with protected areas (PAs), while 552.58 km^2^ (61.67%) are located outside PAs. The total extent of protected areas within the study region is 859.37 km^2^, of which 515.95 km^2^ (60.04%) corresponded to non-habitat land cover. When classified by connectivity importance, 679.40 km^2^ (75.82%) of habitat are categorized as high importance, 149.10 km^2^ (16.64%) as medium importance, and 67.50 km^2^ (7.53%) as low importance. Within protected areas, 281.41 km^2^ (31.41% of total habitat; 81.94% of protected habitat) correspond to high-importance areas, while 46.56 km^2^ (5.20%) and 15.45 km^2^ (1.72%) correspond to medium- and low-importance areas, respectively. Outside protected areas, high-importance habitat accounts for 397.98 km^2^ (44.42% of total habitat), followed by 102.55 km^2^ (11.44%) of medium importance and 52.05 km^2^ (5.81%) of low importance (Fig 6a).

In terms of reliability, 536.45 km^2^ (59.87%) of habitat are classified as low reliability, 246.54 km^2^ (27.51%) as medium reliability, and 113.01 km^2^ (12.62%) as high reliability. Within protected areas, 200.50 km^2^ (22.38% of total habitat) correspond to low-reliability areas, 105.75 km^2^ (11.80%) to medium reliability, and 37.17 km^2^ (4.15%) to high reliability. Outside protected areas, 335.95 km^2^ (37.49%) are classified as low reliability, 140.79 km^2^ (15.71%) as medium reliability, and 75.84 km^2^ (8.47%) as high reliability (Fig 6b).

## Discussion

This study estimates landscape functional connectivity for four seed-dispersing mammals and identifies spatial priorities for conservation in a highly fragmented Andean landscape. Forest cover is limited (remains 18.9% of its original extent) and highly fragmented, resulting in a network dominated by small and isolated patches. This structural configuration is consistent with other human-modified tropical landscapes, where agricultural expansion reduces habitat availability and increases isolation among forest remnants, constraining species movement and ecological processes [3,44]. Consistent with the observed concentration of forest cover above 2,000 m a.s.l., other studies also reported a higher proportion of mature forests in the highland Andes compared to lowland regions in Colombia [45]. This pattern is likely driven by differences in land-use intensity along the elevational gradient. Lower elevations are more accessible and climatically suitable for agriculture and urban development, leading to extensive deforestation and landscape transformation. In contrast, higher elevations are generally less suitable for intensive land use due to steeper slopes, lower temperatures, and logistical constraints, which has limited human pressure and allowed larger areas of forest to persist [46,47].

Functional connectivity values were low across all species, reflecting the scarcity of suitable habitat. This finding aligns with the findings of a national-scale assessment [48], that emphasized that connectivity loss in the Colombian Andes has exceeded the rate of deforestation, with the most severe impacts concentrated in three regions (Eje Cafetero, the southern Andes, and the Cundiboyacense Plateau). Differences in connectivity among species follow expectations from movement ecology, which predicts that species with greater movement capacity and broader environmental tolerance maintain stronger connections between habitat patches [49]. In this study, *A. seniculus*, despite a smaller home range, showed higher connectivity than *C. versicolor*, reflecting its broad altitudinal distribution (up to 3200 m a.s.l.) and ability to exploit a wider range of forest conditions. A similar pattern was observed among rodents. *C. paca* exhibited higher connectivity than *D. punctata*, even though both species have comparable home range sizes, likely due to its wider altitudinal range. These results indicate that dispersal capacity, approximated here by home range size, is not the main driver of connectivity in this system. Instead, environmental tolerance, particularly the ability to occupy a wider elevational gradient, appears to play a dominant role by increasing the amount of accessible habitat and the number of potential connections among patches. This pattern is consistent with findings from studies conducted in fragmented landscapes in the Republic of Korea and Southern California, which demonstrate that species with broader environmental tolerances and access to larger suitable areas tend to maintain higher levels of functional connectivity, even within fragmented systems [50,51].

Understanding these findings is particularly important in Andean ecosystems, where many species are naturally restricted to narrow elevational ranges. As habitat continuity becomes disrupted, populations may become increasingly isolated in “altitudinal islands,” reducing gene flow and ultimately increasing extinction risk. For example, fragmentation of Andean Forest and páramo habitats has isolated spectacled bear (*Tremarctos ornatus*) populations into discrete habitat blocks across the northern Andes, substantially increasing population isolation [52]. In this context, the situation of *C. versicolor* is especially critical. The species is classified as Endangered in the IUCN Red List due to accelerated habitat loss [53]. As an endemic primate with a restricted range, further fragmentation of its habitat in the Andean landscape can quickly intensify isolation between subpopulations, reducing gene flow and increasing the likelihood of local extinctions [53].

The distribution of node importance (dPC) indicates that connectivity is highly uneven, with most habitat nodes contributing minimally (dPC < 1) and a small number accounting for a large share of connectivity. This skewed structure is characteristic of fragmented landscapes, where connectivity depends on a limited set of key habitat patches that function as hubs within the network [54]. The presence of a few nodes with very high dPC values across species suggests that the loss of these patches would lead to a marked reduction in overall connectivity, reinforcing their importance as conservation targets. Species with fewer habitat nodes, such as *C. versicolor*, show a higher individual contribution of each node to overall connectivity, reinforcing the importance of prioritizing conservation strategies in these areas. Incorporating uncertainty provides important context for interpreting these results. High variability in dPC values, particularly for *C.paca*, indicates strong sensitivity to assumptions regarding dispersal distance and matrix resistance. This pattern is likely associated with the large number of habitat nodes identified for this species, resulting in a highly fragmented and spatially complex network in which small changes in resistance values or dispersal thresholds can substantially alter connectivity estimates. Similar patterns have been reported in fragmented landscapes composed of numerous small and isolated habitat patches, where connectivity metrics become strongly dependent on model parameterization [e.g., 55].

Across all species, the flux component dominated dPC, while the connector component was negligible. This indicates that connectivity is primarily maintained through direct dispersal between habitat patches rather than through multi-step stepping-stone pathways. Similar patterns have been reported in highly fragmented systems where inter-patch distances exceed typical dispersal distances, limiting the effectiveness of indirect connections [56]. The limited role of the connector fraction suggests that the current landscape configuration lacks sufficient intermediate patches to support stepping-stone dynamics, a condition often associated with advanced stages of fragmentation [56].

The multi-species prioritization reveals a clear spatial mismatch between connectivity priorities and the existing protected area network. Although protected areas include a proportion of high-connectivity habitat, more than half of the total habitat —and a large fraction of high-priority areas— remain outside formal protection. This gap is consistent with broader assessments in the Andes and other tropical regions, where protected areas are often biased toward less productive or less transformed lands and may not fully capture areas critical for maintaining connectivity [57,58]. These results highlight the importance of complementing existing reserves with conservation strategies in human-dominated landscapes, such as landscape restoration or connectivity-oriented land-use planning [59,60].

The incorporation of uncertainty on the results gives a higher confidence to decision-making frameworks based on spatial models, since not all predicted priority areas have the same level of confidence. In this context, areas identified as high importance with low uncertainty represent robust conservation priorities and should be prioritized for immediate action, whereas areas with high uncertainty should be treated more cautiously, as their contribution to connectivity is less certain and may require additional empirical validation through movement or genetic data before being used to guide management decisions.

From an ecological perspective, reduced connectivity for seed-dispersing mammals has implications beyond species persistence. These taxa play key roles in the dispersal of large-seeded plants, which are often affected by fragmentation due to dispersal limitation [61,62]. Declines in connectivity can therefore disrupt specific plant regeneration processes [61], alter species composition, and ultimately affect forest structure and carbon storage [62]. Maintaining functional connectivity for these mammals is thus closely linked to the resilience and long-term functioning of Andean Forest ecosystems.

## Conclusion

This study provides a clear and integrative picture of how landscape fragmentation is structuring ecological connectivity in the Colombian Coffee Region, revealing that connectivity is extremely low and highly uneven across the landscape, with a small set of habitat patches acting as critical network hubs. A key contribution is demonstrating that connectivity is not primarily driven by dispersal ability or home range size, but by species’ environmental tolerance and elevational breadth, a finding that is especially relevant for montane Andean systems where habitat continuity along elevation gradients is essential. Our work also identifies a strong spatial mismatch between connectivity priorities and the existing protected area network, with most high-priority areas remaining unprotected, highlighting urgent gaps in current conservation strategies. Importantly, by explicitly incorporating uncertainty into multi-species connectivity and prioritization models, the study strengthens decision-making by distinguishing robust conservation priorities from areas requiring further validation. Together, these results provide a robust, spatially explicit approach for conservation planning in fragmented tropical landscapes, and offer critical guidance for maintaining both biodiversity and ecosystem processes such as seed dispersal and forest regeneration.

## Supporting information

S1 Methods Development and parameterization of species-specific resistance surface. (DOCX)

S1 Table Land-cover composition and resistance parameterization used in connectivity analyses. (DOCX)

S2 Table Connectivity model parameterization and resulting connectivity metrics across scenarios. (DOCX)
