# Supplementary material for: Seed disperser connectivity in a heterogeneous landscape of the Colombian Coffee Region
Source: PLoS One. 2026 Jun 26;21(6):e0351834. doi: 10.1371/journal.pone.0351834 (PMC13309012; doi:10.1371/journal.pone.0351834)
Supplement: S1 Methods — (DOCX) [file pone.0351834.s001.docx]

**SUPPORTING INFORMATION**

Restrepo-Carvajal, Clerici & Alvarado. *Seed disperser connectivity in a heterogeneous landscape of the Colombian coffee region*.

**S1 Methods. Development and parameterization of species-specific resistance surface**

Species-specific resistance surfaces were parameterized to represent relative movement costs across land-cover types and anthropogenic features for four seed-dispersing mammals (*Cebus versicolor*, *Alouatta seniculus*, *Cuniculus paca*, and *Dasyprocta punctata*), following good practices in resistance-based connectivity modeling [1]. Resistance values were informed by empirical studies and expert elicitation, and uncertainty was explicitly incorporated through alternative contrast scenarios.

Resistance scenarios were developed using the CORINE Land Cover layer [2] combined with a national road network layer [3], acknowledging the role of roads as pervasive barriers to animal movement through both mortality risk and behavioral avoidance [4,5]. Roads were buffered by 10 m to represent the zone of strongest disturbance, where traffic, noise, and human activity can significantly alter habitat use and movement behavior [4,6].

For primates, resistance assignments reflected their strong dependence on forest cover and sensitivity to fragmentation. *A. seniculus* is a strictly arboreal species that concentrates its activity within forest interiors and rarely crosses open areas, justifying high resistance values for non-forest land covers [7,8]. In contrast, *C. versicolor* shows greater ecological flexibility, using forest edges, secondary vegetation, and occasionally modified matrices under resource limitation, supporting intermediate resistance values for agricultural mosaics and secondary habitats [7,9,10].

For rodents, *C. paca* is strongly associated with dense forest and well-developed understory vegetation, with limited use of open habitats, warranting high resistance values for pastures and bare areas [11]. *D. punctata* tolerates secondary and partially modified habitats but still depends on forest cover for refuge and food resources, supporting moderate resistance values in heterogeneous matrices [12,13]. Experimental studies on scatter-hoarding mammals also support the persistence of seed dispersal processes across partially disturbed landscapes, consistent with non-maximal resistance values in mixed land-cover classes. National-scale species atlases were additionally used to align resistance assignments with documented distributions and habitat suitability patterns in Colombia [14,15].

To complement the literature review, resistance values were refined through expert consultation. Two specialists—a primatologist and a rodent ecologist with extensive experience in Andean ecosystems— were consulted through personal communication. Each expert independently assigned resistance values (1–100 scale) to each land-cover category for each species based on their knowledge of movement behavior, habitat preferences, and responses to disturbance. Final baseline resistance values represent a consensus between literature-based expectations and expert judgment, improving ecological realism and consistency among species.

To explicitly account for uncertainty in resistance parameterization, three alternative resistance scenarios were defined: (1) a low-contrast scenario with reduced differences among land-cover classes, (2) a baseline scenario representing intermediate resistance values, and (3) a high-contrast scenario with stronger resistance assigned to non-forest areas. Evaluating alternative resistance scenarios is a common approach in connectivity modeling to assess the sensitivity of results to uncertainty in resistance assignment and to identify robust connectivity patterns across parameterizations [1,16].

**REFERENCES**

1. Zeller KA, McGarigal K, Whiteley AR. Estimating landscape resistance to movement: a review. Landsc Ecol. 2012;27:777–797. doi:10.1007/s10980-012-9737-0
2. IDEAM. Cobertura de la Tierra Metodología CORINE Land Cover Adaptada para Colombia Periodo 2020 límite administrativo. Bogotá: Instituto de Hidrología, Meteorología y Estudios Ambientales; 2020.
3. Instituto Geográfico Agustín Codazzi. Cartografía vectorial a escala 1:100,000 con cobertura total de la República de Colombia. Bogotá: IGAC; 2022.
4. Forman RTT, Alexander LE. Roads and their major ecological effects. Annu Rev Ecol Syst. 1998;29:207–231. doi:10.1146/annurev.ecolsys.29.1.207
5. Benítez-López A, Alkemade R, Verweij PA. The impacts of roads and other infrastructure on mammal and bird populations: a meta-analysis. Biol Conserv. 2010;143:1307–1316. doi:10.1016/j.biocon.2010.02.009
6. Laurance WF, Goosem M, Laurance SGW. Impacts of roads and linear clearings on tropical forests. Trends Ecol Evol. 2009;24:659–669. doi:10.1016/j.tree.2009.06.009
7. Defler TR. Historia natural de los primates colombianos. Bogotá: Universidad Nacional de Colombia; 2010.
8. Giraldo P, Gómez-Posada C, Martínez J, Kattan G. Resource use and seed dispersal by red howler monkeys (Alouatta seniculus) in a Colombian Andean forest. Neotrop Primates. 2007;14:55–64.
9. Matthews LJ. Activity patterns, home range size, and intergroup encounters in Cebus albifrons support existing models of capuchin socioecology. Int J Primatol. 2009;30:709–728. doi:10.1007/s10764-009-9362-1
10. Montilla D. Actividad y dieta del mono cariblanco (Cebus versicolor) y su relación con la disponibilidad de recursos en un hábitat fragmentado en el Valle del Magdalena Medio, Colombia [thesis]. Armenia: Universidad del Quindío; s.f.
11. Benavides C, Arce A, Pacheco LF. Home range and habitat use by pacas in a montane tropical forest in Bolivia. Acta Amaz. 2017;47:227–236. doi:10.1590/1809-4392201603163
12. Smythe N. Ecology and behavior of the agouti Dasyprocta punctata and related species on Barro Colorado Island, Panama [PhD thesis]. Maryland: University of Maryland; 1970.
13. Aliaga-Rossel E, Kays RW, Fragoso JMV. Home-range use by the Central American agouti (Dasyprocta punctata) on Barro Colorado Island, Panama. J Trop Ecol. 2008;24:367–374. doi:10.1017/S0266467408005085
14. Ramírez-Chaves HE, Cruz-Rodríguez CA, Noguera-Urbano EA, Gutiérrez C. Atlas de la Biodiversidad de Colombia: grandes roedores. Bogotá: Instituto Humboldt; 2022.
15. Olaya-Rodríguez MH, Noguera-Urbano EA, Gutiérrez C. Atlas de la biodiversidad de Colombia: primates. Bogotá: Instituto Humboldt; 2020.
16. Spear SF, Balkenhol N, Fortin MJ, McRae BH, Scribner K. Use of resistance surfaces for landscape genetic studies: considerations for parameterization and analysis. Mol Ecol. 2010;19:3576–3591. doi:10.1111/j.1365-294X.2010.04657.x
