# Supplementary material for: Seed disperser connectivity in a heterogeneous landscape of the Colombian Coffee Region
Source: PLoS One. 2026 Jun 26;21(6):e0351834. doi: 10.1371/journal.pone.0351834 (PMC13309012; doi:10.1371/journal.pone.0351834)
Supplement: S1 Table — (DOCX) [file pone.0351834.s002.docx]

**SUPPORTING INFORMATION**

Restrepo-Carvajal, Clerici & Alvarado. *Seed disperser connectivity in a heterogeneous landscape of the Colombian coffee region*.

**S2 Table. Land-cover composition and resistance parameterization used in connectivity analyses.** Land-cover categories included in the study, their total area (km²) and proportional representation within the study area, and resistance values assigned to each class for each species under baseline, low-contrast, and high-contrast scenarios.

* Forest area includes edge habitats and all forest patches, including those smaller than the minimum home range of any species.

| **Land Cover** | **Area km^2^** | **Area %** | **Baseline scenario** | | | | **Low contrast scenario** | | | | **High contrast scenario** | | | |
| --- | --- | --- | --- | --- | --- | --- | --- | --- | --- | --- | --- | --- | --- | --- |
|  |  |  | ***Cebus versicolor*** | ***Alouatta seniculus*** | ***Cuniculus paca*** | ***Dasyprocta punctata*** | ***Cebus versicolor*** | ***Alouatta seniculus*** | ***Cuniculus paca*** | ***Dasyprocta punctata*** | ***Cebus versicolor*** | ***Alouatta seniculus*** | ***Cuniculus paca*** | ***Dasyprocta punctata*** |
| Industrial and urban area | 172.70 | 2.74 | 100 | 100 | 100 | 100 | 80 | 80 | 80 | 80 | 1000 | 1000 | 1000 | 1000 |
| Road | 144.00 | 2.29 | 100 | 100 | 100 | 100 | 80 | 80 | 80 | 80 | 1000 | 1000 | 1000 | 1000 |
| Mining | 1.32 | 0.02 | 100 | 100 | 100 | 100 | 80 | 80 | 80 | 80 | 1000 | 1000 | 1000 | 1000 |
| Transitional crops | 21.63 | 0.34 | 70 | 90 | 50 | 40 | 50 | 60 | 40 | 35 | 200 | 300 | 120 | 100 |
| Permanent herbaceous crops | 50.91 | 0.81 | 70 | 90 | 50 | 40 | 50 | 60 | 40 | 35 | 200 | 300 | 120 | 100 |
| Permanent shrub crops | 408.58 | 6.49 | 20 | 20 | 40 | 30 | 25 | 25 | 35 | 30 | 40 | 40 | 80 | 60 |
| Permanent tree crops | 106.47 | 1.69 | 20 | 20 | 40 | 30 | 25 | 25 | 35 | 30 | 40 | 40 | 80 | 60 |
| Pastures | 1293.31 | 20.55 | 70 | 80 | 60 | 50 | 50 | 60 | 45 | 40 | 300 | 400 | 200 | 150 |
| Mosaic of pastures and/or crops | 984.48 | 15.64 | 70 | 70 | 50 | 40 | 50 | 50 | 40 | 35 | 250 | 250 | 120 | 100 |
| Mosaic of natural spaces. crops and/or pastures | 1271.74 | 20.20 | 50 | 40 | 40 | 30 | 40 | 35 | 35 | 30 | 120 | 80 | 80 | 60 |
| Forest (Habitat nodes) | 1190.84* | 18.92* | 1 | 1 | 1 | 1 | 1 | 1 | 1 | 1 | 1 | 1 | 1 | 1 |
| Forest edges (30m) | - | - | 5 | 5 | 5 | 5 | 10 | 10 | 10 | 10 | 15 | 15 | 15 | 15 |
| Forested areas without suitable habitat (smaller area than home range) | - | - | 5 | 5 | 5 | 5 | 10 | 10 | 10 | 10 | 20 | 20 | 20 | 20 |
| Forest plantation | 223.53 | 3.55 | 30 | 20 | 40 | 30 | 35 | 30 | 40 | 35 | 80 | 60 | 120 | 80 |
| Grassland | 2.66 | 0.04 | 70 | 70 | 50 | 40 | 50 | 50 | 40 | 35 | 250 | 250 | 120 | 100 |
| Bushland | 8.24 | 0.13 | 60 | 50 | 40 | 30 | 45 | 40 | 35 | 30 | 180 | 150 | 80 | 60 |
| Secondary vegetation | 373.83 | 5.94 | 20 | 20 | 20 | 20 | 25 | 25 | 25 | 25 | 40 | 40 | 40 | 40 |
| Sandy areas and bare lands | 1.56 | 0.02 | 80 | 90 | 60 | 70 | 60 | 65 | 50 | 55 | 400 | 500 | 250 | 300 |
| River | 37.72 | 0.60 | 60 | 50 | 50 | 60 | 45 | 40 | 40 | 50 | 150 | 120 | 120 | 150 |
| Artificial bodies of water | 0.90 | 0.01 | 70 | 60 | 60 | 70 | 50 | 50 | 45 | 50 | 200 | 150 | 150 | 200 |
