# Supplementary material for: Seed disperser connectivity in a heterogeneous landscape of the Colombian Coffee Region
Source: PLoS One. 2026 Jun 26;21(6):e0351834. doi: 10.1371/journal.pone.0351834 (PMC13309012; doi:10.1371/journal.pone.0351834)
Supplement: S2 Table — (DOCX) [file pone.0351834.s003.docx]

**SUPPORTING INFORMATION**

Restrepo-Carvajal, Clerici & Alvarado. *Seed disperser connectivity in a heterogeneous landscape of the Colombian coffee region*.

**S2 Table. Connectivity model parameterization and resulting connectivity metrics across scenarios.** Table includes matrix resistance scenarios, dispersal percentiles and their associated weights, estimated and effective dispersal distances (m and m·cost, respectively), mean landscape resistance, and resulting connectivity values expressed as Probability of Connectivity (PC) and Equivalent Connected Area (ECA, km²) for each species and scenario.

| **Species** | **Matrix scenarios** | **Dispersal percentil** | **Dispersal Weight** | **Dispersal distance m** | **Mean resistance** | **Effective dispersal distance** | **PC** | **ECA km^2^** |
| --- | --- | --- | --- | --- | --- | --- | --- | --- |
| *Alouatta seniculus* | Baseline | P95 | 0.2 | 7563 | 45.92 | 347269 | 0.0011497 | 213.5 |
| *Alouatta seniculus* | Baseline | P75 | 0.25 | 3664 | 45.92 | 168239 | 0.0007684 | 174.6 |
| *Alouatta seniculus* | Baseline | P50 | 0.25 | 2214 | 45.92 | 101660 | 0.0006282 | 157.9 |
| *Alouatta seniculus* | Baseline | P25 | 0.15 | 1337 | 45.92 | 61391 | 0.0005523 | 148.0 |
| *Alouatta seniculus* | Baseline | P10 | 0.1 | 850 | 45.92 | 39029 | 0.0005230 | 144.0 |
| *Alouatta seniculus* | High contrast | P95 | 0.2 | 7563 | 201.41 | 1523261 | 0.0022137 | 296.3 |
| *Alouatta seniculus* | High contrast | P75 | 0.25 | 3664 | 201.41 | 737965 | 0.0013999 | 235.6 |
| *Alouatta seniculus* | High contrast | P50 | 0.25 | 2214 | 201.41 | 445921 | 0.0010630 | 205.3 |
| *Alouatta seniculus* | High contrast | P25 | 0.15 | 1337 | 201.41 | 269285 | 0.0008445 | 183.0 |
| *Alouatta seniculus* | High contrast | P10 | 0.1 | 850 | 201.41 | 171198 | 0.0007085 | 167.6 |
| *Alouatta seniculus* | Low contrast | P95 | 0.2 | 7563 | 37.54 | 283934 | 0.0009817 | 197.3 |
| *Alouatta seniculus* | Low contrast | P75 | 0.25 | 3664 | 37.54 | 137556 | 0.0006857 | 164.9 |
| *Alouatta seniculus* | Low contrast | P50 | 0.25 | 2214 | 37.54 | 83119 | 0.0005814 | 151.9 |
| *Alouatta seniculus* | Low contrast | P25 | 0.15 | 1337 | 37.54 | 50194 | 0.0005316 | 145.2 |
| *Alouatta seniculus* | Low contrast | P10 | 0.1 | 850 | 37.54 | 31911 | 0.0005155 | 143.0 |
| *Cebus versicolor* | Baseline | P10 | 0.1 | 1472 | 51.68 | 76070 | 0.0000173 | 17.5 |
| *Cebus versicolor* | Baseline | P25 | 0.15 | 2317 | 51.68 | 119738 | 0.0000196 | 18.6 |
| *Cebus versicolor* | Baseline | P50 | 0.25 | 3834 | 51.68 | 198134 | 0.0000236 | 20.4 |
| *Cebus versicolor* | Baseline | P75 | 0.25 | 6346 | 51.68 | 327949 | 0.0000302 | 23.1 |
| *Cebus versicolor* | Baseline | P95 | 0.2 | 13100 | 51.68 | 676983 | 0.0000469 | 28.8 |
| *Cebus versicolor* | High contrast | P10 | 0.1 | 1472 | 212.01 | 312072 | 0.0000227 | 20.0 |
| *Cebus versicolor* | High contrast | P25 | 0.15 | 2317 | 212.01 | 491217 | 0.0000265 | 21.6 |
| *Cebus versicolor* | High contrast | P50 | 0.25 | 3834 | 212.01 | 812830 | 0.0000330 | 24.1 |
| *Cebus versicolor* | High contrast | P75 | 0.25 | 6346 | 212.01 | 1345388 | 0.0000431 | 27.6 |
| *Cebus versicolor* | High contrast | P95 | 0.2 | 13100 | 212.01 | 2777275 | 0.0000689 | 34.9 |
| *Cebus versicolor* | Low contrast | P10 | 0.1 | 1472 | 40.85 | 60133 | 0.0000160 | 16.8 |
| *Cebus versicolor* | Low contrast | P25 | 0.15 | 2317 | 40.85 | 94652 | 0.0000177 | 17.7 |
| *Cebus versicolor* | Low contrast | P50 | 0.25 | 3834 | 40.85 | 156623 | 0.0000205 | 19.1 |
| *Cebus versicolor* | Low contrast | P75 | 0.25 | 6346 | 40.85 | 259241 | 0.0000252 | 21.1 |
| *Cebus versicolor* | Low contrast | P95 | 0.2 | 13100 | 40.85 | 535148 | 0.0000371 | 25.6 |
| *Cuniculus paca* | Baseline | P10 | 0.1 | 269 | 43.97 | 11828 | 0.0000974 | 48.5 |
| *Cuniculus paca* | Baseline | P25 | 0.15 | 423 | 43.97 | 18600 | 0.0000984 | 48.8 |
| *Cuniculus paca* | Baseline | P50 | 0.25 | 700 | 43.97 | 30780 | 0.0001022 | 49.7 |
| *Cuniculus paca* | Baseline | P75 | 0.25 | 1159 | 43.97 | 50963 | 0.0001116 | 51.9 |
| *Cuniculus paca* | Baseline | P95 | 0.2 | 2392 | 43.97 | 105180 | 0.0001397 | 58.1 |
| *Cuniculus paca* | High contrast | P10 | 0.1 | 269 | 156.83 | 42188 | 0.0001047 | 50.3 |
| *Cuniculus paca* | High contrast | P25 | 0.15 | 423 | 156.83 | 66341 | 0.0001151 | 52.8 |
| *Cuniculus paca* | High contrast | P50 | 0.25 | 700 | 156.83 | 109784 | 0.0001348 | 57.1 |
| *Cuniculus paca* | High contrast | P75 | 0.25 | 1159 | 156.83 | 181770 | 0.0001734 | 64.7 |
| *Cuniculus paca* | High contrast | P95 | 0.2 | 2392 | 156.83 | 375146 | 0.0003637 | 93.8 |
| *Cuniculus paca* | Low contrast | P10 | 0.1 | 269 | 36.63 | 9853 | 0.0000972 | 48.5 |
| *Cuniculus paca* | Low contrast | P25 | 0.15 | 423 | 36.63 | 15493 | 0.0000976 | 48.6 |
| *Cuniculus paca* | Low contrast | P50 | 0.25 | 700 | 36.63 | 25639 | 0.0000996 | 49.1 |
| *Cuniculus paca* | Low contrast | P75 | 0.25 | 1159 | 36.63 | 42450 | 0.0001059 | 50.6 |
| *Cuniculus paca* | Low contrast | P95 | 0.2 | 2392 | 36.63 | 87611 | 0.0001277 | 55.6 |
| *Dasyprocta punctata* | Baseline | P10 | 0.1 | 269 | 39.12 | 10523 | 0.0000119 | 9.2 |
| *Dasyprocta punctata* | Baseline | P25 | 0.15 | 423 | 39.12 | 16548 | 0.0000121 | 9.3 |
| *Dasyprocta punctata* | Baseline | P50 | 0.25 | 700 | 39.12 | 27384 | 0.0000127 | 9.5 |
| *Dasyprocta punctata* | Baseline | P75 | 0.25 | 1159 | 39.12 | 45340 | 0.0000143 | 10.1 |
| *Dasyprocta punctata* | Baseline | P95 | 0.2 | 2392 | 39.12 | 93576 | 0.0000194 | 11.8 |
| *Dasyprocta punctata* | High contrast | P10 | 0.1 | 269 | 146.89 | 39513 | 0.0000128 | 9.6 |
| *Dasyprocta punctata* | High contrast | P25 | 0.15 | 423 | 146.89 | 62134 | 0.0000142 | 10.1 |
| *Dasyprocta punctata* | High contrast | P50 | 0.25 | 700 | 146.89 | 102823 | 0.0000169 | 11.0 |
| *Dasyprocta punctata* | High contrast | P75 | 0.25 | 1159 | 146.89 | 170245 | 0.0000211 | 12.3 |
| *Dasyprocta punctata* | High contrast | P95 | 0.2 | 2392 | 146.89 | 351361 | 0.0000308 | 14.8 |
| *Dasyprocta punctata* | Low contrast | P10 | 0.1 | 269 | 34.52 | 9286 | 0.0000118 | 9.2 |
| *Dasyprocta punctata* | Low contrast | P25 | 0.15 | 423 | 34.52 | 14601 | 0.0000119 | 9.2 |
| *Dasyprocta punctata* | Low contrast | P50 | 0.25 | 700 | 34.52 | 24163 | 0.0000123 | 9.4 |
| *Dasyprocta punctata* | Low contrast | P75 | 0.25 | 1159 | 34.52 | 40007 | 0.0000132 | 9.7 |
| *Dasyprocta punctata* | Low contrast | P95 | 0.2 | 2392 | 34.52 | 82569 | 0.0000168 | 11.0 |
